# ErpC, a member of the complement regulator-acquiring family of surface proteins from *Borrelia burgdorferi*, possesses an architecture previously unseen in this protein family

**DOI:** 10.1107/S1744309113013249

**Published:** 2013-05-23

**Authors:** Joseph J. E. Caesar, Steven Johnson, Peter Kraiczy, Susan M. Lea

**Affiliations:** a Sir William Dunn School of Pathology, University of Oxford, South Parks Road, Oxford OX1 3RE, England; b Institute of Medical Microbiology and Infection Control, Frankfurt University Hospital, Paul-Ehrlich-Strasse 40, 60596 Frankfurt, Germany

**Keywords:** BbCRASP-4, *Borrelia burgdorferi*, ErpC, factor H, complement

## Abstract

*Borrelia burgdorferi* is a spirochete responsible for Lyme disease, the most commonly occurring vector-borne disease in Europe and North America. The bacterium utilizes a set of proteins, termed complement regulator-acquiring surface proteins (CRASPs), to aid evasion of the human complement system by recruiting and presenting complement regulator factor H on its surface in a manner that mimics host cells. Presented here is the atomic resolution structure of a member of this protein family, ErpC. The structure provides new insights into the mechanism of recruitment of factor H and other factor H-related proteins by acting as a molecular mimic of host glycosaminoglycans. It also describes the architecture of other CRASP proteins belonging to the OspE/F-related paralogous protein family and suggests that they have evolved to bind specific complement proteins, aiding survival of the bacterium in different hosts.

## Abstract 2

The structure of ErpC, a member of the complement regulator-acquiring surface protein family from *B. burgdorferi*, has been solved, providing insights into the strategies of complement evasion by this zoonotic bacterium and suggesting a common architecture for other members of this protein family.

## 1. Introduction

*Borrelia burgdorferi* is a Gram-negative spirochete which, following transmission into the dermis during feeding of an infected *Ixodes* tick, may result in Lyme borreliosis, the most commonly occurring vector-borne disease in Europe and North America (Centers for Disease Control and Prevention, 2007 ▶; Steere, 1989 ▶; Steere *et al.*, 2004 ▶). The predominant indications of infection include a spontaneously resolving skin rash (erythema migrans) often accompanied by other symptoms including headache and fever (Stanek & Strle, 2003 ▶; Steere, 1989 ▶). A chronic multisystemic disorder can result if the infection is not immediately cleared by host immunity or antibiotic treatment, allowing spirochetes to spread to multiple organs within the host (Steere, 1989 ▶).

*Borrelia* species have developed multiple strategies for evading the different immune systems across their range of reservoir hosts, which include the capture and presentation of host complement regulators, a mechanism that is also exhibited by many pathogenic bacteria (Embers *et al.*, 2004 ▶; Lambris *et al.*, 2008 ▶; Zipfel *et al.*, 2007 ▶). The resistance of distinct *Borrelia* species towards the complement response upon exposure to human serum has been linked to the binding of the major alternative-pathway regulators factor H and factor-H-like protein-1 (FHL-1) by a family of molecules termed complement regulator-acquiring surface proteins (CRASPs; Kraiczy, Skerka, Brade *et al.*, 2001 ▶; Kraiczy, Skerka, Kirschfink *et al.*, 2001 ▶; Stevenson *et al.*, 2002 ▶; Kraiczy & Stevenson, 2013 ▶).

Factor H is a 155 kDa protein consisting of 20 short consensus-repeat (SCR) domains, of which the four N-terminal domains possess decay-accelerating activity towards the alternative-pathway C3 convertase and act as a cofactor for factor I-mediated cleavage of C3b (Pangburn *et al.*, 1977 ▶; Vik *et al.*, 1990 ▶; Whaley & Ruddy, 1976 ▶). Although circulating in the blood, the local concentration of factor H is increased on self-cell surfaces *via* interactions with glycosamino­glycans that are characterized by heparin-binding sites found in domains 6 and 7 and 19 and 20 (Prosser *et al.*, 2007 ▶; Schmidt *et al.*, 2008 ▶). Bacteria have been shown to bind factor H in these regions using surface-protein glycosaminoglycan mimics (Schneider *et al.*, 2009 ▶).

To date, the only atomic resolution structure of a member of the CRASP family is that of CspA (also referred to as CRASP-1, BbCRASP-1 or BBA68) from *B. burgdorferi* (Cordes *et al.*, 2005 ▶). CspA is a 26 kDa protein that possesses a predominantly α-helical secondary structure and forms a homodimeric species which is necessary for factor H binding (Cordes *et al.*, 2006 ▶). Following *in vitro* mutagenesis studies, a putative factor H binding site has been proposed within the cleft between the two subunits which interacts with factor H and FHL-1 in the region of domains 5–7 (Kraiczy *et al.*, 2004 ▶, 2009 ▶). Another member of the CRASP family, ErpC (also referred to as BbCRASP-4), is an 18 kDa protein that belongs to the OspE/F-related (Erp) paralogous family of proteins and has also been demonstrated to bind factor H (Kraiczy, Skerka, Brade *et al.*, 2001 ▶; Kenedy & Akins, 2011 ▶). Moreover, ErpC has been implicated in the scavenging of complement factor H-related proteins CFHR-1, CFHR-2 and CFHR-5 (Haupt *et al.*, 2007 ▶; Kraiczy, Skerka, Brade *et al.*, 2001 ▶; Hammerschmidt *et al.*, 2012 ▶), although the functional implications of this ability to bind multiple complement proteins has yet to be fully understood. Presented here is the atomic resolution structure of ErpC and analyses that provide further insights into the mechanisms of the binding of complement proteins by *B. burgdorferi*.

## 2. Experimental

### 2.1. Expression and purification of selenomethionine-derivatized protein

The generation of a plasmid expressing ErpC with deletion of the hydrophobic leader-encoding sequence (residues 1–20) and with an N-­terminal glutathione-*S*-transferase (GST) purification tag has been described previously (Haupt *et al.*, 2007 ▶). *Escherichia coli* strain B834(DE3) transformed with this plasmid was cultured in SelenoMet medium (Molecular Dimensions) supplemented with 40 mg l^−1^ l-­seleno­methionine at 310 K prior to induction of expression of the fusion protein by the addition of 1 m*M* isopropyl β-d-1-thiogalactopyranoside during mid-log phase and continued incubation at 294 K for 18 h. The GST-ErpC fusion was purified from cell-lysate supernatant using a GSTrap FF column (GE Healthcare) as per the manufacturer’s instructions prior to removal of the purification tag by incubation with HRV-3C protease (Novagen). A final purification step was performed using a Superdex 75 column (GE Healthcare) pre-equilibrated in 50 m*M* Tris, 150 m*M* NaCl, 10 m*M* β-mercapto­ethanol, 1 m*M* ethylenediaminetetraacetic acid pH 7.5.

### 2.2. Crystallization, data collection and processing

Crystals of ErpC were obtained after 24 h from a 1:1(*v*:*v*) mixture of a stock solution of selenomethionine-derivatized ErpC (*A*_280_ = 10.5) and 27%(*w*/*v*) PEG 2000 MME, 0.1 *M* sodium cacodylate pH 6.5 at 294 K using vapour diffusion in 800 nl sitting drops produced by an Oryx Nano crystallization robot (Douglas Instruments). The crystals were backsoaked in 20% ethylene glycol, 27%(*w*/*v*) PEG 2000 MME, 0.1 *M* MES pH 6.5 for approximately 3 s prior to cryocooling and data collection at the Se *L*_III_ absorption maximum as described in Table 1 ▶. Data were processed using *xia*2 (Winter, 2010 ▶) invoking the 3da flag to enforce usage of *XDS* (Kabsch, 2010 ▶) and *AIMLESS* (Evans, 2006 ▶). Data-processing statistics are reported in Table 1 ▶.

### 2.3. Structure determination and refinement

The ErpC structure was solved using Se-SAD from one selenomethionine residue in each of the two copies of the protein in the asymmetric unit. The *autoSHARP* phasing pipeline (Vonrhein *et al.*, 2007 ▶) was used for structure solution, using *SHELXD* (Sheldrick, 2008 ▶) for site finding and *SHARP* (Bricogne *et al.*, 2003 ▶) for heavy-atom site refinement followed by solvent flattening with *SOLOMON* (Abrahams & Leslie, 1996 ▶; Winn *et al.*, 2011 ▶). The final overall figures of merit were 0.48 and 0.13 for acentric and centric reflections, respectively, whilst the overall phasing power was 1.17. An initial model was built from the experimentally phased map using *Buccaneer* (Winn *et al.*, 2011 ▶) prior to iterative rounds of refinement in *autoBUSTER* (Blanc *et al.*, 2004 ▶; Bricogne *et al.*, 2011 ▶) and rebuilding using *Coot* (Winn *et al.*, 2011 ▶; Emsley *et al.*, 2010 ▶). The final coordinates were validated using the *MolProbity* server (Chen *et al.*, 2010 ▶) and deposited in the PDB with accession code 4bf3. Refinement and validation statistics are reported in Table 2 ▶.

### 2.4. Multi-angle laser-light scattering

100 µg of sample was injected onto a Superdex 200 10/300 column (GE Healthcare) and the elution was monitored using a Dawn Helios II (Wyatt Technology) and an Optilab T-rEX (Wyatt Technology) to measure the scattering and the refractive index, respectively. All data were analysed using *ASTRA* (Wyatt Technology).

## 3. Results

**Figure 1:**
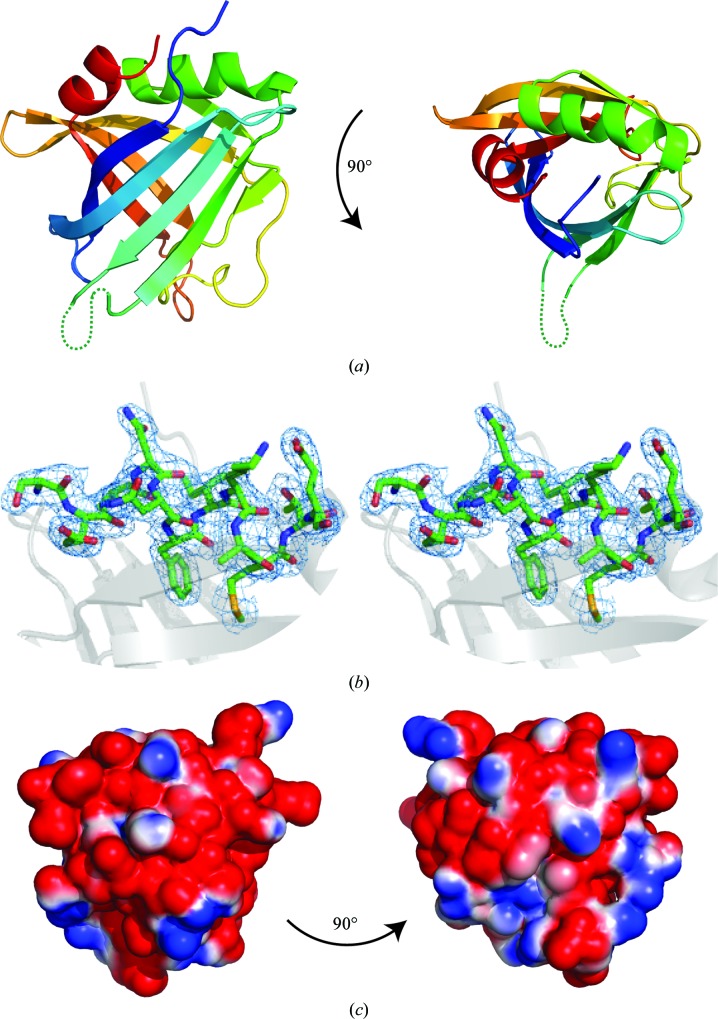
(*a*) Views of the atomic resolution structure of ErpC with the secondary structure shown in cartoon representation. The main chain is coloured from the N-terminus (blue) to the C-terminus (red). Loops that were not observed in the crystal structure are represented as dotted lines. This figure was generated using *PyMOL* v.1.5.0.4 (Schrödinger). (*b*) Stereoview of representative electron density around the N-terminal α-helix (residues 99–110). A 2*F*_o_ − *F*_c_ σ_A_-weighted map contoured at 0.1353 e Å^−3^ is shown. (*c*) Representations of the charge density on the surface of ErpC calculated using *APBS* (Baker *et al.*, 2001 ▶).

**Figure 2:**
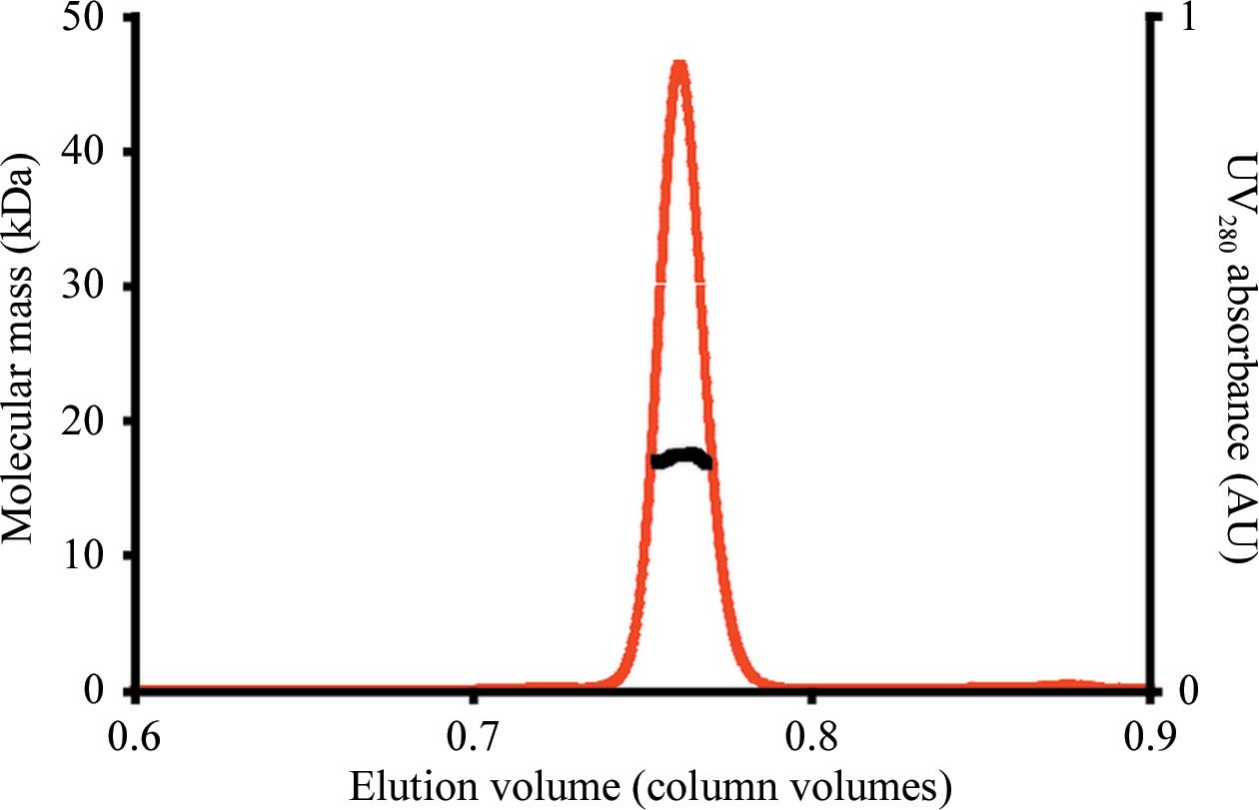
Multi-angle laser-light scattering analysis of ErpC in solution. The mass distribution of ErpC (molecular mass 18 316 Da) is monodisperse, consistent with its existence in a monomeric form in solution.

The structure of ErpC has been solved, revealing an architecture consisting of ten antiparallel β-strands forming a barrel capped by two α-helices (Fig. 1 ▶ *a* and 1 ▶ *b*). The molecule possesses a hydrophobic core, whilst the outer surface is highly charged (Fig. 1 ▶ *c*). Several of the loops between β-strands could not be built in one or both copies of ErpC in the unit cell owing to a lack of electron density, suggesting that these regions are conformationally labile. A search for structurally similar proteins using the *PDBeFold* protein-structure comparison service at the European Bioinformatics Institute (http://www.ebi.ac.uk/msd-srv/ssm; Krissinel & Henrick, 2004 ▶) revealed high levels of secondary-structure similarity to a sporulation-specific cell-division protein from *Thermobifida fusca*, SsgB (PDB entry 3cm1; Xu *et al.*, 2009 ▶; r.m.s.d. of 2.3 Å), and to two mitochondrial RNA-binding proteins from *Trypanosoma brucei*, MRP1 and MRP2 (PDB entry 2gid; Schumacher *et al.*, 2006 ▶; r.m.s.d.s of 3.0 and 2.9 Å, respectively). SsgB was observed to form a homotrimer, whilst MRP1 and MRP2 form a heterotetramer. However, ErpC lacks the additional structural elements that are involved in these assemblies, suggesting that it does not multimerize in a similar manner, and analysis of the interfaces between the ErpC molecules in the crystal using *PISA* (Krissinel & Henrick, 2007 ▶) suggested that there were no biologically relevant assemblies. Multiple-angle laser-light scattering was used to investigate the oligomeric state of ErpC in solution. The observed molecular mass of 17 000 Da correlated with the expected molecular mass of ErpC (18 316 Da), confirming that the protein is monomeric under these conditions (Fig. 2 ▶) and, in conjunction with the *PISA* analysis, that it is likely to be monomeric on the spirochete surface.

## 4. Discussion

ErpC is a member of the CRASP family of proteins which aid in complement evasion by *B. burgdorferi* by binding and presenting complement factor H on the bacterial cell surface under distinct circumstances (Kenedy & Akins, 2011 ▶). The atomic resolution structure of ErpC has been solved, showing the formation of a β-­barrel, an architecture which is completely different to the pre­dominantly α-helical CspA, which is the only other member of the CRASP family for which the structure has been elucidated (Cordes *et al.*, 2005 ▶). However, both ErpC and CspA have been reported to bind factor H, suggesting that the functions of these molecules have evolved separately (Kraiczy, Skerka, Brade *et al.*, 2001 ▶). This is further confirmed by the observation that ErpC is monomeric both in solution and in the crystal lattice, suggesting that this is its functional state, while CspA forms a homodimeric species which is necessary for factor H binding (Cordes *et al.*, 2005 ▶).

Factor H is localized on self-cell surfaces *via* interaction with glycosaminoglycans (Meri & Pangburn, 1990 ▶) and it has previously been demonstrated that bacterial species have evolved protein mimics of these highly charged molecules in order to recruit this complement regulator in a manner analogous to host cells (Schneider *et al.*, 2009 ▶). The solvent-accessible surface of ErpC exhibits large regions of negative charge, which suggests that ErpC may be acting in a similar capacity by binding factor H *via* interaction interfaces reserved for host-cell localization.

**Figure 3:**
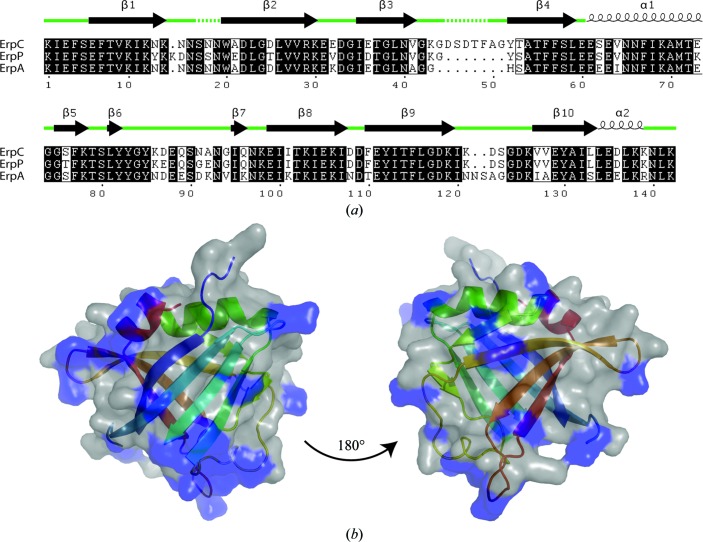
(*a*) ErpC, ErpP and ErpA possess a common architecture. Sequence alignments of ErpC with ErpP and ErpA show high levels of conservation within the secondary-structure elements of ErpC (shown above), suggesting that all three proteins have the same fold. Sequence differences occur mainly in the loop regions between β-strands, suggesting that these regions may have evolved to bind specific complement proteins. Loop regions observed in the ErpC crystal structure are highlighted by a continuous green line. Those which were not observed are shown by a dotted green line. (*b*) Mapping of sequence similarity onto the structure of ErpC. Sequence differences between ErpC, ErpP and ErpA highlighted in (*a*) are coloured in blue on the surface representation of ErpC.

The binding site of complement regulators on ErpC is likely to involve residues within the loops between the β-strands in a similar manner to other bacterial factor H-binding proteins (Schneider *et al.*, 2009 ▶). ErpC shares a high level of sequence similarity with two other CRASP proteins belonging to the OspE/F-related paralogous protein family, namely ErpP (also known as BbCRASP-3) and ErpA (also known as BbCRASP-5), with identities of approximately 65 and 69%, respectively. Mapping the sequence conservation between ErpC, its paralogue OspE and members of the OspE/F-related protein family, including ErpP and ErpA, across different *B. burgdorferi* strains reveals that variation occurs mainly within these loops (Fig. 3 ▶). These findings suggest that ErpC, ErpP, ErpA and OspE share a common architecture and are individually tailored to binding specific complement proteins with different affinities. The evolution of separate proteins with these functions may be key to the survival of *B. burgdorferi* in a specific host or may aid in complement evasion across the range of reservoir hosts of the zoonotic spirochete.

These findings may also explain why ErpC has also been observed to bind members of the factor H-related family of proteins in addition to factor H. However, the functional rationale behind the binding of these proteins has yet to be fully understood. The binding of CFHR1, CFHR2 and CFHR5 by ErpC is further complicated by the observation that these proteins exist in both homodimeric and heterodimeric forms (Goicoechea de Jorge *et al.*, 2013 ▶). ErpC may bind one of these proteins in a specific manner, but the presence of the others as a result of heterodimerization may previously have been interpreted as binding. Further investigation of these interactions may reveal an even greater specificity of ErpC for binding specific factor H-related proteins.

*Note added in proof:* Since submission of this manuscript the structure of the ErpC paralogue OspE in complex with factor H domains 19 and 20 has been published (Bhattarcharjee *et al.*, 2013 ▶).

## Supplementary Material

PDB reference: ErpC, 4bf3

## Figures and Tables

**Table 1:** Data-collection and processing statistics Values in parentheses are for the highest resolution shell.

Diffraction source	I03, Diamond
Detector	PILATUS 6M-F
Temperature (K)	120
Wavelength (Å)	0.979
Space group	*P*2_1_2_1_2_1_
Unit-cell parameters (Å)	*a* = 62.4, *b* = 68.0, *c* = 75.4
No. of molecules in unit cell *Z*	8
Matthews coefficient *V*_M_ (Å^3^ Da^−1^)	2.21
Solvent content (%)	44.5
Resolution (Å)	24.15–2.37 (2.46–2.37)
*R*_merge_ †	0.074 (0.778)
〈*I*/σ(*I*)〉	13.7 (2.6)
Completeness (%)	99.7 (99.9)
Average multiplicity	5.3 (5.3)
Wilson *B* factor (Å^2^)	56.4
*f*′ (e)	−8.59
*f*′′ (e)	6.48

† *R*_merge_ = 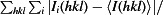 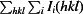.

**Table 2:** Structure refinement and model validation Values in parentheses are for the highest resolution shell.

Refinement software	*autoBUSTER*
Refinement on	*F*
Resolution (Å)	24.15–2.37 (2.56–2.37)
No. of reflections	13623 (2712)
No. of reflections for *R*_free_	675 (160)
*R*_work_/*R*_free_	0.19/0.23 (0.21/0.22)
No. of atoms	
Protein	2099
Ligand/ion	60 [ethylene glycol]
Water	24
Mean *B* factors (Å^2^)	
Protein	56.2
Ligand/ion	59.9 [ethylene glycol]
Water	48.6
R.m.s. deviations from ideal values†	
Bond lengths (Å)	0.01
Bond angles (°)	1.18
Ramachandran plot analysis‡, residues in	
Most favoured regions (%)	97.59
Disallowed regions (%)	0.4
Clashscore§	1.42
Poor rotamers‡ (%)	3.48

† As reported by Engh & Huber (1991 ▶).

‡ Statistics calculated using *MolProbity* (Chen *et al.*, 2010 ▶).

§ Clashscore represents the number of steric overlaps (>0.4 Å) per 1000 atoms.
